# ‘The living death of Alzheimer's’ versus ‘Take a walk to keep dementia at bay’: representations of dementia in print media and carer discourse

**DOI:** 10.1111/1467-9566.12122

**Published:** 2014-08-01

**Authors:** Elizabeth Peel

**Affiliations:** Institute of Health and Society, University of Worcester

**Keywords:** dementia, Alzheimer's disease, media, carers, discourse

## Abstract

Understanding dementia is a pressing social challenge. This article draws on the ‘Dementia talking: care conversation and communication’ project which aims to understand how talk about, and to people living with dementia is constructed. In this article I draw on the construction of dementia manifest in two data sets – a corpus of 350 recent UK national newspaper articles and qualitative data derived from in-depth interviews with informal carers. These data were analysed using a thematic discursive approach. A ‘panic-blame’ framework was evident in much of the print media coverage. Dementia was represented in catastrophic terms as a ‘tsunami’ and ‘worse than death’, juxtaposed with coverage of individualistic behavioural change and lifestyle recommendations to ‘stave off’ the condition. Contrary to this media discourse, in carers' talk there was scant use of hyperbolic metaphor or reference to individual responsibility for dementia, and any corresponding blame and accountability. I argue that the presence of individualistic dementia ‘preventative’ behaviour in media discourse is problematic, especially in comparison to other more ‘controllable’ and treatable chronic conditions. Engagement with, and critique of, the nascent panic-blame cultural context may be fruitful in enhancing positive social change for people diagnosed with dementia and their carers.

## Introduction

Dementia is an increasingly common disease, which predominantly affects older people. Globally there is estimated to be 25 million people living with dementias (Ferri *et al*. 2005) and in the UK over 800,000 people (Alzheimer's Society 2012); 64,000 of whom are under the age of 65 (Luengo-Fernandez *et al*. 2010). The economic cost to the National Health Service, local authorities and families is £23 billion a year; more than cancer and heart disease combined (Alzheimer's Society 2013a, Luengo-Fernandez *et al*. 2010). Current projections suggest that over 1 million people in Britain will be living with dementia by 2021 given trends towards an ageing population (Alzheimer's Society 2012). Understanding dementia is, therefore, a pressing challenge as societal awareness increases (McParland 2012), the economic cost of care is recognised (Knapp *et al*. 2007) and calls for a redress of the legacy of research underfunding amplify (Luengo-Fernandez *et al*. 2010). Dementias, the most common of which are Alzheimer's disease, vascular dementia and fronto-temporal dementia, are progressive – ultimately terminal – diseases that affect memory, communication, mood and behaviour. Currently there is no cure for any type of dementia and the evidence-base for the efficacy of the available pharmacological treatments is not particularly strong. Understandably, then, fear of getting dementia is prevalent (Corner and Bond 2004) and there is much work to be done to ameliorate the stigma and negative connotations associated with Alzheimer's disease and dementias more broadly (Batsch and Mittelman 2012, Jolley and Benbow 2000). As Greening *et al*. (2009: 20) intimate, ‘the prospect of failing memory and the differentiation between normal consequences of ageing and the more serious difficulties of dementia or Alzheimer's disease is perhaps the most feared’.

In the UK, there has been a recent surge in governmental interest in prioritising dementia (Department of Health 2009) with attendant monetary commitment from the current coalition government to address the ‘national crisis’ posed by dementia. Public awareness was identified as a central focus of the £13 million Economic and Social Research Council/National Institute of Health Research dementia initiative launched in July 2012. The popular media is an important cultural site that both creates and reflects public awareness of health issues. As Lupton (1999: 260) has argued:

For many lay people, the mass media constitute one of the most important sources of information about health and medicine. Mass media portrayals contribute to the creation or reproduction of knowledges about illness and disease … they work to portray ill people in certain lights (for example, as ‘innocent victims’ or ‘deserving of their fate’).

Invariably our understandings about different diseases, and those who live with them, are constructed by, as well as mediated through, the discourses promulgated in the media (Clarke 2006, Collin and Hughes 2011, Lyons 2000, Seale 2002, Williamson *et al*. 2011). It has been claimed that ‘dominant constructions of health and illness in the media may function to influence how people behave with regard to their own, and others', health’ (Lyons 2000: 351). Therefore, the media is an important site to scrutinise not only in terms of the constructions of events it provides but also in terms of its potential impacts on different groups and individuals.

Only a very small number of articles, to date, have examined the representation of dementia in the media in Canada and the USA (Clarke 2006, Kang *et al*. 2010) and in New Zealand (Kirkman 2006) or compared coverage across the USA, Canada and the UK (Adelman and Karlawish 2003). Juanne Clarke (2006) inductively analysed 25 articles about Alzheimer's disease printed in the top 20 highest circulation magazines in Canada in the years 1991, 1996 and 2001. Alison Kirkman (2006) analysed 1327 articles from 15 New Zealand newspapers from 1996 to 2002. Kang *et al*.'s (2010) sample was 1372 transcripts of US newscasts and news segments in information talk shows from 1984 to 2008. While dementia, particularly Alzheimer's disease, has not attracted the degree of media analysis that cancer has (for example, Clarke and Everest 2006, Musso and Wakefield 2009) the topic has been positioned as having a higher media-value than other conditions, such as hypertension (Collin and Hughes 2011). Some commentators have gone as far to suggest that dementia is ‘over-expressed by the print media’ (Williamson *et al*. 2011: 549). This observation, however, was derived from a quantitative analysis of volume of media coverage and tells us little about the construction or content of media representations of dementia. The established position is that health issues relevant to older people are less frequently discussed in the media (Seale 2002: 115). A number of themes have emerged from the existing analyses of media coverage of dementia and Alzheimer's disease specifically. Kirkman (2006), for example, outlined the emphasis on the biomedical model in scientific reports; the anticipation of actual and social death; personal stories derived from carers rather than people with dementia themselves (see also Clarke 2006); tales of negligence in care homes and missing people reports. Taken together, it has been suggested that the ‘cultural representations of people with dementia … are essentially negative’ (Bond *et al*. 2004: 220–1). Yet the nuance of contemporary media representations of dementia and the salience (or not) of media coverage to those caring for people with dementia, has yet to be explored. Bringing together one form of cultural representation of dementia – in this case newspaper coverage – with data from individuals caring for a person with dementia forms one lens, among many,^1^ through which the research question ‘how is dementia represented in society?’ can be explored.

In this article I argue that there are two predominant and paradoxical discursive representations of dementia. These are encapsulated in the title of this article, which is taken from headlines in two British newspapers the *Daily Mail* (‘The living death of Alzheimer's’, Revoir 2011) and the *Express* (‘Take a walk to keep dementia at bay’, Fletcher 2010). The latter discourse relates both to a current focus on ‘living well’ with dementia (Department of Health 2009) and growing biomedical emphasis on the vascular – and therefore more ‘controllable’ – aspects of dementias, and is also a logical extension of broader (western) liberal-humanistic discourses of self-responsibility for health and wellbeing (Lyons 2000). The former is a panic or catastrophising discourse, which has a longer history in dementia studies and associates living with dementia as a form of social death (Sweeting and Gilhooly 1997). As Sweeting and Gilhooly (1997) argued based on their interview study of 100 carers of people with dementia, ‘society may view or treat the dementia sufferer as a liminal or non-person, who is demonstrably making the transition from life to death’ (p. 99). The phrase ‘death-in-life’ was used by clinical psychologist Robert Kastenbaum to describe Alzheimer's disease and other dementias (Kastenbaum 1988), and we see that notion represented here in this recent newspaper headline.

I combine discussion of these discourses in the British newspaper print media with talk about cultural representations of dementia derived from interviews with carers of people with dementia to argue that, although individual responsibility for dementia is gaining momentum in media reporting, carers are either unaware of, or resistant to notions of blame and accountability for dementia. My aim in so doing is, following Clarke (2006: 274), to contribute to ‘the development of a critical perspective on the taken-for-granted truths and realities’ about dementia and those living with dementia. This analysis also contributes to broader discussion with respect to the question posed by sociologist Nikolas Rose (2006: 84):

Where are the boundaries between conditions for which an individual is to be accorded responsibility, and those for which responsibility is to be located elsewhere – in the organs, in fate, in heredity?

In line with the proliferation of self-responsibility discourses more generally, the boundaries with respect to the onset of dementia appear to be in flux and (certainly at the level of media representation) may – as I suggest though the following analysis – be foregrounding individual responsibility in new, and potentially troubling, ways.

## Methodology

The overarching theoretical, and ideological, frame for this analysis is that provided by critical health psychology, which inheres attentiveness to the social, political and cultural dimensions of health and illness and a commitment to promoting an improved quality of life in vulnerable and marginalised groups (Lyons 2000, Murray 2004). I take a broadly social constructionist approach to the newspaper coverage, which as Riggs (2005: 238) suggests, involves moving beyond a focus either on the production or reception of media to a focus on the ideologies that underpin particular media representations. My analysis is based on British print media collected over a 1-year period (1 October 2010 to 30 September 2011). This time-frame was chosen for a number of reasons. First, it reflects my emphasis and interest on contemporary cultural representations of dementia. Second, it is some time after the publication, in February 2009, of the *National Dementia Strategy,* a landmark document on the government's vision and policy for people living with dementia and dementia services (Department of Health 2009). Third, it is a long enough time period to include key dates connected to the issue, such as World Alzheimer's Day (21 September), Dementia Awareness Week and the inaugural Dementia Awareness Day (17 September), where heightened levels of media coverage might be expected. In line with my previous media analyses (Jowett and Peel 2010), the focus is on the print media specifically because, in comparison with other forms of media the press is interesting and contradictory, as its owned and controlled by those with power but claims to represent and articulate the views of the people (Conboy 2002).

The LexisNexis database, which contains text web versions of national and regional newspapers plus trade and industry magazines from around the world, was used to access the newspaper articles. The database was searched using the terms ‘dementia’ or ‘Alzheimer's’ both in the headline and as ‘major mentions’ in the text of UK national newspapers only. This initial search resulted in 2540 hits. In order, therefore, to create a broad but manageable data set for analysis I narrowed the search to national newspapers that contained either ‘dementia’ or ‘Alzheimer's’ in the headline alone. (*The Sun* has the highest circulation figure of these newspapers, at just over 3 million and the *Daily Telegraph* has the highest readership of the broadsheets at 650,000 [Luft 2011]). All the headlines (*n* = 499) were screened to remove repetition and irrelevant articles (for example, tangential arts reviews), which resulted in a final data set of 350 articles. In terms of the spread of articles across the newspapers: 38 per cent were located in ‘mid-market’ tabloids; 34 per cent in broadsheets; and 28 per cent were in red-top tabloids. The *Daily Telegraph* and *The Times* offered the most broadsheet coverage while the *Express*, the *Mirror* and the *Sunday Mirror* contained the most tabloid coverage. The tone and content of the media coverage was largely the same across the newspapers examined, although there was lexical variation between the tabloid and broadsheet press (for example, ‘obesity’ in *The Guardian* and ‘fatties’ in the *Mirror*).

Following university ethical approval, the informal carers of people with dementia were recruited via their participation in an earlier pilot study focusing on carers' experiences of the regulation of, and access to, dementia care (Peel and Harding 2013). The recruitment strategy for this study involved third sector organisations (for example, Dementia UK) advertising the study, and is described in detail elsewhere (see, Harding and Peel 2013, Peel and Harding 2013). Table 1 provides information about the 12 carers (two of whom were interviewed jointly) interviewed for the ‘Dementia talking’ project, the overall aim of which is to understand how talk about and to people with dementia is constructed with the (ultimate) goal of improving communication with people with dementia. The project aim and the research questions (see Dementia Project n.d.) were provided for carers in the information sheet; and the topic areas of representation of dementia in society, how dementia is talked about, communication with people with dementia and communicative strategies and approaches were covered in the interview schedule.

**Table 1:** Participants' characteristics

Pseudonym	Age	Class	Person cared for/age	Dementia type	Residence of PWD	Caring status
Victoria	63	Middle	Mother, 88	Alzheimer's	Own home	Current
Carlos and Anne	58	Working	Father, 87	Alzheimer's	Own home	Ex-carer
Jan	58	Working	Mother, 87	Vascular dementia	Residential home	Current
Emma	79	Middle	Husband, 83	Vascular dementia	Residential home (self-funding)	Current
Sue	59	Working	Mother, 87	Vascular dementia	Residential home (self-funding)	Current
Derek	65	Working	Mother, 86	Vascular dementia	Own home	Ex-carer
Maureen	60	Middle	Mother, 95	Alzheimer's	Nursing home (self-funding)	Ex-carer
Kaylet	59	Working	Husband, 67	Fronto-temporal dementia	Own home	Current
Jonathan	67	Middle	Wife, 66	Fronto-temporal dementia	Nursing home (NHS continuing care)	Current
Mick	70	Working	Wife, 68	Alzheimer's	Nursing home (NHS continuing care)	Current
Pamela	56	Middle	Husband, 60	Fronto-temporal dementia	Own home	Current

The interviews ranged from 1 hour 16 minutes to 2 hours 7 minutes (mean length 1 hour 37 minutes) and were conducted in the participants' homes between November 2011 and January 2012. Most interviews were conducted in the Midlands, three were conducted in the north of England and two in the south. All participants were white and most identified as heterosexual, with the exception of one bisexual woman. The mean age of the interviewees was 63 years (range 56–79) and the mean age of the person they cared for was 79.5 years (range 60–95). The carers were asked one main question about cultural representations of people with dementia followed by prompts and a follow-up question: Do you have any thoughts about how people with dementia are represented in society? (in television programmes, newspapers, documentaries and so on); and what do you think about how people with dementia and the illness are talked about in society? The intention of these questions was not to understand their engagement with print media specifically (that is, what newspapers, if any, they read), or to investigate their views about specific media coverage. Rather, the purpose was to explore the carers' general perspectives on the nature, tone and scope of societal representations of dementia and people living with dementia. Responses to these questions were collated and the transcripts were read and coded for any further talk about societal and/or media representation of dementia across the 11 interviews. Similarly, the newspaper articles were repeatedly read and systematically coded following the approach outlined by Braun and Clarke (2006). The thematic approach used to interrogate the media and interview data was theoretically informed by the discursive psychology discourse analytic tradition (Potter and Wetherell 1987), which emphasises a social constructionist stance on language and the ideological and political implications of particular discourses and discursive strategies. In the analysis that follows I examine the two overarching discourses encapsulated by the headlines ‘The living death of Alzheimer's’ and ‘Take a walk to keep dementia at bay’ comparing, and contrasting, discussion of the newspaper articles with talk from the interviews with carers.

## Catastrophising, ‘panic’ and the dementia epidemic discourse

In these newspaper data there was a preponderance of headlines emphasising the catastrophic nature of dementia in various ways. For example, ‘Cancer and Alzheimer's most feared of diseases’ (*Morning Star* 2011), ‘Dementia “A bomb ready to explode”; Warning’ (*Mirror* 2011) and ‘Brain disease on rise; Alzheimer's epidemic’ (Hamilton 2010) construct dementia as a threat or imminent catastrophe. Of course, the notion of health-related epidemics are not unfamiliar in both academic and lay discourses with respect to diabetes (Peel *et al*. 2005), obesity (Rich 2011) and an ageing demographic in western countries generally. But it is interesting to see the demographic time-bomb metaphor being deployed with respect to dementia specifically, which (at least in part) functions to bring an immediacy and urgency to dementia as a public health issue. Dementia is characterised in ways that emphasise the ‘horror’ of the condition – such as ‘a terrible affliction’ (*Express* 2011); a ‘crippling brain-wasting disease’ (*Sunday Mirror* 2011) – and its progressivity: ‘brain-wasting’, ‘harrowing descent into Alzheimer's’ (Bates 2011).

Extract 1 is a typical exemplar of the nature and content of this discourse, also explicitly highlighted in phrases such as ‘dementia is our most pressing, and costly, medical and social problem’ (*Daily Telegraph* 2010).

### Extract 1: Media

Comment: Alzheimer's is compelling in fiction – and so cruel in life: It is not glib to impute significance from the disease's hold in popular culture – we now fear it more than death itself.

… it is the particular character of this condition – its merciless assault on memory, the locus of selfhood and our connection with others – as much as its prevalence that renders it compelling in fiction and so dreaded in reality. People fear dementia more than cancer and even more than death, according to a poll undertaken earlier this year…. Those working in the sector contend that, in terms of public awareness, research and funding, it remains 30 years behind a disease of similar social impact. Cancer research received £590m funding last year, compared to the £50m devoted to dementia, despite a study finding that the economic effect of dementia was greater than that of cancer and heart disease combined. Perhaps this time lag will give the media the opportunity to employ a little more nuance, thus avoiding the usual panic-blame approach evident in the reporting of atypical tales of younger onset and faddy avoidance tactics. (Brooks 2011: 42)

Brooks' characterisation of dementia's ‘hold in popular culture’ draws on the Alzheimer's Research Trust and the University of Oxford's health economics report on the economic burden of dementia and the paucity of research funding in the area to make some interesting observations, about both fictional and cultural perceptions of dementia, and the media's own engagement with the topic (Luengo-Fernandez *et al*. 2010). This report was picked up and publicised widely across the print media, including the *Daily Telegraph* (2010) (‘research into dementia remains woefully underfunded’) and the *Mirror*. The foregrounded issue of fear of dementia, known as anticipatory dementia (Cutler and Hodgson 1996) or dementia worry (DW) in the literature, resonates with the ‘emerging evidence that DW is a relatively widespread and probably increasing phenomenon in Western societies and that DW is at the top of all disease worries’ (Kessler *et al*. 2012). In *The Guardian* excerpt in Extract 1, Brooks constructs the particular character of dementia in ways that bolster the contemporary cultural focus on fear of dementia through phrases such as ‘merciless assault’, ‘dreaded’ and ‘cruel’. This example is also interesting in that it offers a form of meta-commentary on the media's engagement with the topic of dementia, described as ‘the usual panic-blame approach’. As we see later, in the analysis of the individual responsibility for health discourse evident in these media data, the phrase ‘faddy avoidance tactics’ is rather an apt way of encapsulating the media portrayal of biomedical research in this area.

The comparison in Extract 1 between dementia and cancer was resonant, too, in carers' accounts. For instance, Derek,^2^ who had cared for his mother in her home until her death, constructed the contrast as:

[I]t's difficult to say which is the worst, cancer or dementia. I mean, you can't compare them really, but dementia, Alzheimer's, it's an absolutely wicked illness. Wicked. Wicked for the person that's suffering, and wicked for the carer that's looking after that person. Terrible, terrible thing, it is.

Through claiming that the two diseases are incomparable, then using the contrastive conjunction ‘but’, Derek positions dementia as ‘worst’ by describing and reiterating that it is ‘wicked’ – which invokes a sense of evilness or sinfulness – and ‘terrible’. In extract 2, Anne, who alongside her husband cared for her father-in-law until his death, draws on a comparison with cancer in a way that is more similar to that of Brooks in *The Guardian* excerpt above.

### Extract 2: Carer

There's been individual stories, like Terry Pratchett, you know, and– and so on, and I think that's quite helpful for people to see that this is such a clever man who's now got this part of his brain that doesn't work anymore. And I think awareness is– is a big part of it because I– I think we're at with dementia where we were with cancer 20 years ago. Twenty years ago people didn't really know that much about– you heard people dying of it and so on but there wasn't the research and the awareness and the campaigns on television that it can happen to whoever. (Anne)

By comparing dementia to cancer ‘20 years ago’ Anne highlights both the seriousness of the illness and also echoes the contemporaneous media emphasis on research and awareness. A second comparator that appeared in the media data, but *not* the carer's data, was with HIV/AIDS: ‘Britain faces dementia catastrophe; Alzheimer's disease should be tackled as aggressively as AIDS was in the Eighties, warn charities’ (Beckford 2011). When HIV/AIDS emerged in the 1980s it was conceptualised as a ‘plague’ and a contagion (Kitzinger and Peel 2005, Seale 2002, Sontag 1988); something that required an immediate and concerted public health intervention. Dementia, by contrast, is neither new (having been identified by Alois Alzheimer in 1907), contagious nor straightforwardly preventable.

But ‘charities,’ in drawing this comparison, engender a sense of immediacy and crisis to the situation regarding dementia and also imply the lack of a known cure for dementias, which mirrors the historical situation of HIV/AIDS. The new, frightening and terminal disease that HIV/AIDS was 20 years ago is now – at least in countries where anti-viral medications are affordable and accessible – transformed into a manageable chronic condition. However, the comparison between the two, potent as it may be on some levels, collapses when we reflect on the solution to the epidemic. The solution to the spread of HIV was/is uncontaminated blood products and safe sex, but there is no simple solution to the prevention of dementias, given the primary biomedical risk factor is understood to be increasing age. If we were to follow this logic then the ‘solution’ to the ‘dementia catastrophe’ would be to cull older people, or actively lead unhealthy lives in an attempt to maximise the chances of dying younger in order to circumvent the possibility of developing dementia, neither of which are thinkable, let alone advisable, in advanced liberal democracies. However, as we go on to see in the individualised responsibility for health discourse a version of the solution is very much a product of advanced liberal democracies ‘where individuals are enjoined to think of themselves as actively shaping their life course’ (Rose 2006: 26). Interestingly, although the carers mentioned individual celebrities with dementia being more visible in the media, such as Terry Pratchett (Ann, Derek, Pamela), Iris Murdoch (Emma, Pamela), Glen Campbell and Norman Wisdom (Derek), they did not typically discuss the broader characterisation of dementia as a public health catastrophe. Only Sue, who cared for her mother who was currently in a residential care setting, drew on the demographic time bomb metaphor. This was the only instance in the carer interviews where the hyperbolic metaphors prevalent in the newspaper data were used.

### Extract 3: Carer

It's the time bomb, isn't it, you know, we're all going to get it because we're going to live– if we live long enough we're all going to get it because simple biology, you're going to get dementia. So it's ‘Oh my God’. It's like something to be frightened of that's coming down the path … I think it's talked about in quite a negative way, as if it's a terrifying– I mean it is frightening in a way when you've got it– it must be terrifying, because my mum says, ‘I'm losing my mind’. (Sue)

Here, Sue highlights the fact that dementia is ‘talked about in quite a negative way’, and uses the evocative words ‘frightening’ and ‘terrifying’. She also alludes to the demographic inevitability of developing dementia in old age and uses active voicing and an exclamation of shock (‘Oh my God’) to emphasise the immediacy and personal impact that this population-level discourse can have on individuals. She then ties this observation about media and broader cultural discourse back to her own situation with her mother by linking the generic with the personal tragedy of dementia, but tempers this (‘in a way’) and links this to her mother's verbalised insight about her dementia, but not to her own position. Sue's account is very different from Buckland's (2010) emotive (and alliterative) description: ‘As it tears sufferers' lives apart, the disease also unleashes a devastating domino effect through families as they watch their loved ones slip away’.

Overall, this aspect of the print media coverage emphasises the epidemic proportion and significant societal impact of dementia. In a further illustration, in the *Daily Telegraph* (Smith 2010) the head of the Alzheimer's Society is quoted as saying ‘dementia represents a ticking time bomb’ and in the same article a ‘dementia suffer’ is quoted as saying ‘there is a dementia tsunami coming’. Dementia is firmly represented as ‘the scourge of the 21^st^ century, a silent epidemic that sneaks up on unsuspecting victims’ (Barry 2010), in these print media. As will become apparent in the analysis of the second prevailing discourse, this contrasts somewhat paradoxically with the strong media emphasis on individual responsibility.

## ‘Prevention’, individual responsibility and blame discourse

The notion that dementia sneaks up on unsuspecting victims and therefore is an insidious and indiscriminate invader of older people's bodies is antithetical to another prevalent discourse in these media data. The blame discourse coheres around constructing dementia onset ‘as a function of individual behaviour’ (Lyons 2000: 353–4). This discourse is encapsulated in headlines such as ‘Worried you'll get Alzheimer's? Then follow these seven steps' (Laurance 2011) and ‘Living a healthier life can halve risk from Alzheimer's’ (Reynolds 2011), which represent dementia as avoidable through lifestyle changes and individual behavioural practices. In this newspaper coverage these suggestions largely focus on intellectual stimulation, drinking and eating, weight and physical activity. In an article in *The Times* entitled ‘Dementia: your questions answered; Brain power Who gets dementia, how can you avoid it and what are the treatments’, for instance, the rhetorical question ‘How can I avoid it?’ is posed and answered with ‘You can't avoid the risk entirely because of the link with age. But you can radically improve your chances' (Carlyle 2010). Here, then, is the bold assertion (‘radically’) that the onset of dementia can be evaded by behaviour that an individual can engage in. Previous media research in this area has suggested that the disease is ‘presented as inevitable for those who live to grow old’ (Clarke 2006: 272). While this is still present in the contemporary British print media, as apparent in the first part of the Carlyle's answer to the rhetorical question about avoiding onset, the suggestion that it can be ‘staved off’ by lifestyle modifications is also ascendant. I suggest that, although a culturally prevalent discourse about individual responsibility for health and illness is not new (Lyons 2000), the emphasis on self-responsibility is new in this context. Similarly, in Extract 4, from a health article in *The Guardian* entitled ‘Dr Luisa Dillner's guide to … Signs of dementia’ (Dillner 2010):

### Extract 4: Media

What are the risk factors?

Getting older and having people in your family who have had dementia increases your own risk. Having high blood pressure increases your risk of vascular dementia as does drinking heavily and smoking.

Can it be prevented?

There is some evidence that if you are mentally and physically active and if you get out of the house, have friends you socialise with, read, play an instrument, take courses, play bridge or golf, or walk regularly, you are less likely to develop dementia.

In the description of risk factors the hereditary component of (some forms) of Alzheimer's disease is implied but not made explicit. This article is one of very few, in these media data, to differentiate between Alzheimer's dementia and vascular dementia. The description of risks in Extract 4 is rather different from the message communicated in other articles that ‘nobody knows what causes the disease’ (for example, Hamilton 2010). But more interesting is the presentation of a range of activities, some of which are very particular – golf, bridge – which mean ‘you are less likely to develop dementia’. Thus a contrast, and arguably conflict, is constructed between the immutability of (some of) the risks – ageing and heritability – and the putative ‘ease’ of prevention. Similarly, an article in *The Times* (Naish 2010) states that: ‘Research shows that people who don't regularly challenge themselves intellectually through work or learning are far more likely to suffer from dementia in later life’. This makes reference to what is colloquially referred to as ‘use it or lose it’ but is couched, as we see here, in more authoritative terms (‘research shows’).

In terms of food and drink much of the coverage is predicated on biomedical research about the composition of particular micronutrients, for example, ‘A vitamin B pill a day may ward off Alzheimer's’ (MacRae 2011), ‘Vitamin C curbs dementia’ (*Express* 2011) or ‘Beetroot can fight dementia’ (*Express* 2010). While these headlines were largely found in the tabloid press they were also covered in the broadsheets. For instance, the potentially beneficial properties of green tea are mentioned: ‘Tea cheers; green leaves fight dementia’ (Armstrong 2011), ‘Tea fights dementia’ (*The Sun* 2011) and ‘Drinking green tea may help ward off Alzheimer's’ (*The Guardian* 2011). Although the headline here in *The Guardian* is somewhat more measured (‘may’) than in the tabloids the term ‘fight’ is especially prevalent. There was also a range of stories about moderate alcohol consumption being beneficial in this context: ‘Moderate drinking ‘can protect against dementia’’ (Hope 2011a); ‘A tipple ‘fights dementia’’ (Alleyne 2011) ‘A pint of beer a day may keep dementia away’ (*Daily Telegraph* 2011). Contradictory health information about dietary composition was also evident in this 1-year print media sample: ‘Can a low-fat diet stave off Alzheimer's?’ (Borland 2011) and ‘Stick to a high-fat diet to avoid Alzheimer's, say researchers’ (*Daily Telegraph* 2010).

*The Times*, in an article entitled ‘What to eat to help prevent Alzheimer's; stock up on the food, vitamins and drinks that can reduce the risks of dementia by Jean Carper’ (Carper 2011a), reprinted an excerpt from Carper's (2011b) self-help book *100 Simple Things You Can Do to Prevent Alzheimer's and Age-Related Memory Loss*. The material consists of 20 didactic health behaviour messages, some of which constitute generic healthy eating advice, such as ‘beware of bad fats’ and others that are specifically related to dementia ‘prevention’, such as:

Drink apple juice. Apple juice can boost the production of acetylcholine in the brain, which is what the number-one-prescribed pharmaceutical drug Aricept (donepezil) does to treat Alzheimer's, according to recent research. Eat two apples or have two cups of juice per day. (p. 8)

Although none of the carer interviewees talked about messages about diet and dementia onset, it is interesting to consider what the impact might be of such materials. An online review of Carper's book highlights the potential impact these individualised health behaviour change messages could have, on, for instance, carers of a biological parent:

With a parent suffering from this dreaded disease I thought I would welcome a book that informs and helps me avoid risk factors. In fact, the very opposite happened … This is a book that can put the fear of god into you and makes it seem like you will follow that familial pathway to dementia … every page is almost like scaremongering. (Taylor 2012)

Carper's book is one example of the growing dementia prevention self-help literature that purports to ‘show you how to strengthen your brain, prevent and treat disease’ (Cook 2007, back cover), ‘fight back’ against ‘an epidemic of stumbling minds’ (Victoroff 2004: 10) and claims that ‘memory decline, dementia and even Alzheimer's are preventable’ (Holford 2011: 1).

Regarding weight, the coverage hinged on one main story about obesity in middle age, typified in headlines including: ‘Weight gain link with dementia’ (*Daily Telegraph* 2011), ‘Losing that spare tyre may help you avoid Alzheimer's’ (Hope 2011b), ‘How staying slim slashes risk of dementia’ (Willey 2011a), ‘Obesity in middle age raises dementia risk by 300%’ (Jha 2011) and ‘Fatties 80% more likely to develop Alzheimer's’ (Buckland 2011). Media coverage of the benefits of physical activity was more broadly spread through these data and encompassed a range of stories on this topic.

### Extracts 5: Media

Daily stroll helps ward off dementia, says study. (*Daily Telegraph* 2011)

How a little exercise in middle age could help beat dementia. (Willey 2011b)

Rake some leaves to cut dementia risk. (*Daily Mail* 2011)

Keep fit to reduce the risk of dementia. (Cannon 2011)

Take a walk and prevent Alzheimer's. (Fletcher 2010)

Why walking nine miles a week could save you from dementia. (Wainwright 2010)

Stroll stops Alzheimer's. (*Mirror* 2010)

We can see in all these headlines focusing on preventing dementia through lifestyle and health behaviour modifications the recurrence of terms such as fight, ward off, stave off, beat, save, stop, reduce, avoid and prevent. These terms unambiguously present the onset of dementia as controllable through individual actions. An interesting comparator here is with media and lay representations of type 2 diabetes, which although in many ways very different to dementia, is also a progressive condition associated with older, and increasing, age. From a biomedical perspective the onset of type 2 diabetes is caused by a range of risk factors including a genetic predisposition, a high fat or sugar diet, low levels of physical activity and being overweight (Lawton *et al*. 2008); in other words, a multifactorial aetiology leaning towards lifestyle factors. Evidence from analysis of type 2 diabetes media coverage in the USA in 2005 and 2006 indicates that the predominant explanation for type 2 diabetes is behavioural and lifestyle factors and obesity, and that a key approach to addressing diabetes is individualised behaviour changes (Gollust and Lantz 2009). In this more recent dementia media coverage the emphasis on prevention and individual responsibility for dementia onset or avoidance of the risk of developing dementia in later life echoes the representations of type 2 diabetes – a condition whereby individual behaviours can and do enable ‘control’ of the disease (Parry *et al*. 2006). Dementia onset and development is arguably far less within the ‘control’ of individuals than type 2 diabetes, if at all.

Carers, on the whole, did not talk about the cultural proliferation of preventative lifestyle and health behaviour related to dementia. Emma, who was caring for her husband who was residing in a nursing home, was the exception.

### Extract 6: Carer

You keep reading things like, well, make sure that you keep active, that you keep reading, that you do the crossword and, well Tim did the crossword [laughs] every day of his life, it didn't stop him getting dementia. So there's an implication there that it's somehow your fault if you get dementia because you're not being active enough or doing the crossword or, you know, whatever um, and it– it doesn't really make any difference at all whether Tim had done the crossword or not, he would have still got dementia. And who knows whether it's genetic or– we– we don't know, you know, what it is, there's still not enough– there's a lot of ignorance about it still. (Emma)

Here we see Emma creating a sense of the strength of the presence of these health-related messages by repeating ‘you keep reading’. She then constructs the notion that there are activities that can prevent the onset of dementia as didactic commands (‘well, make sure you keep active’ and so on), reminiscent of the way, as we have seen, these messages are conveyed in print media. She then contrasts this with her own, opposing, experience. She adds weight to her experiential authority by using the extreme case formulation ‘every’ in reporting that her husband, Tim ‘did the crossword every day of his life, it didn't stop him getting dementia’. She then goes on to directly link these individual lifestyle recommendations with blame and personal culpability for the development of dementia – ‘there's an implication there that it's somehow your fault if you get dementia’ – before talking about the lack of knowledge about the aetiology of dementia. Therefore, we can see in Emma's talk that blame and a sense of personal responsibility for dementia onset (and in this instance, resistance to those embedded presumptions) may be the effect of the emerging discourse that emphasises individual responsibility for dementia prevention via engagement in certain lifestyle or health-related behaviour. Indeed, Rose's (2006: 25) work on biomedicine and biopolitics claims that:

This is an ethic in which the maximisation of lifestyle, potential, health, and quality of life has become almost obligatory, and where negative judgements are directed toward those who will not, for whatever reason, adopt an active, informed, positive, and prudent relation to the future.

While the newspaper stories did not connect lifestyle and health advice with individual culpability (aside from Brooks' [2011] mention of a media ‘panic-blame approach’ in *The Guardian*) and neither did most of the carers, that it was articulated by Emma could signal an emerging additional level of stigmatisation of people living with dementia and their carers. While these individualised health messages may have valence for the ‘worried well,’ the impact on those already diagnosed with a dementia – especially vascular dementia,^3^ which was the case for Emma's husband – and their families may be detrimental.

## Discussion

Two discourses about dementia were identified – one related to notions of a dementia ‘epidemic’, the other health and lifestyle factors associated with the ‘prevention’ of dementia. The first discourse is underpinned by a biomedical ideology that firmly positions dementia as a disease and pathology not in any way connected to ‘normal’ ageing. It has been claimed that ‘most lay people believe that significant memory loss is an inevitable consequence of aging’ (Bonnesen and Burgess 2004: 136) and this association has been identified in previous analyses of media coverage of dementia (Kirkman 2006). However, the media data analysed here draw a sharp distinction between ageing *per se* and the scale and impact of dementia as a public health ‘crisis’; making very explicit the notion that dementias are progressive and fatal diseases and not merely forgetfulness in older age. The identification of the second prevention discourse also extends the existing literature, and signals an individualising and potentially victim-blaming turn in lay understandings of dementias. As Rich (2011: 5) notes ‘imperatives around ‘eating well’, exercising regularly and monitoring our bodies, carry powerful moral overtones about how individuals ought to behave’. These imperatives have not, however, been previously identified with respect to dementia onset or, more precisely, dementia prevention. The potential shift identified in this analysis connects to arguments in biopolitics in that is it acknowledged that:

Technologies of government have involved an increasing emphasis on the responsibility of individuals to manage their own affairs … nowhere have these been more telling than in the field of health. (Rose 2006: 4)

This responsibilisation is especially troubling in the dementia arena, given inadequacies in biomedical solutions, the lack of cure, or of effective treatments and, I would argue less optimistically than Rose (2006: 27), a largely absent ‘moral economy of hope’, despite the current rhetoric of living well with dementia (Department of Health 2009).

From a critical health psychology perspective the discourse identified in these print media materials represent a worrying foregrounding of the cultural representation of the causes of dementias as preventable (as opposed to unknown and unpreventable) through lifestyle and behavioural modifications that do not operate at a public health level, but rather individualise responsibility for the development of an incurable and terminal disease. This may potentially be to the detriment of people diagnosed with dementia and their families. Again, to quote Rose (2006: 26–7):

[O]f course an ethic organised around the ideals of health and life produces anxiety, fear, even dread at what one's biological future, or that of those one cares for, might hold.

Given that only one of these carers discussed the blaming connotations of ‘the use it or lose it’ idiom and just one other used the hyperbolic metaphor of the time bomb, it may be that the impacts of these discourses are insidious and invisible. Certainly, examining if, and in what ways, these constructions of dementia differentially impact on various groups and closer lay media readings would be a fruitful avenue of enquiry. This could be explored with respect to groups including people living with early-stage dementia, people with young-onset dementia, the worried well or people with functional memory difficulties that do not have an organic basis, relatives with (and without) a biological connection to a person with dementia, health-care and social-care professionals and so on. Doubtless the complex nexus of increased public profile of dementia and anticipatory dementia or dementia worry warrants further scrutiny. And it would be particularly interesting to explore how people living with dementia interpret these panic and (potentially) blame discourses – are they seen as salient, are they perceived as generating nihilism or hope, or increased stigmatisation?

Unfortunately the visual images and pictures associated with newspaper stories are not available for analysis in LexisNexis. There has been a suggestion that images associated with dementia in the German media may be more positive (Kessler and Schwender 2012) than the ‘prevailing image in post modernity of the social incompetence of people with dementia’ (Bond *et al*. 2004: 223). This may provide another productive avenue of enquiry. Future research could also analyse the dementia causation accounts of both people living with dementia and their carers and families in order to interrogate them for media influence in terms of aetiology, prevention and lifestyle modifications (Lawton *et al*. 2007, 2008). Moreover, media analyses could take a more genealogical approach and chart the enduring, and shifting, cultural representations of dementia and those living with dementia across media sources and other social sites over a much longer period. There was little evidence in 2010–2011 newspaper coverage of an increase in the presence of people living with dementia themselves in stories on the topic, except for the occasional activist voice alongside those of representatives of charitable organisations such as the Alzheimer's Society. Clarke's (2006) analysis of coverage from 1991–2001 highlighted the missing person with dementia in media representation, and this seems to have endured. However, to my knowledge, a focus on preventative behaviour is a recent phenomenon; and one that is particularly problematic in the dementia context when compared to, say, type 2 diabetes or other controllable chronic conditions. Understanding the impacts this discourse does and undoubtedly will increasingly have on different individuals and groups, and how it may be challenged or reclaimed will be important in generating positive social change for people living with dementia (as opposed to the worried well) and their carers.

## Notes

1 In line with a broadly social constructionist approach I acknowledge that many forms of data could have been brought to bear in exploring the contemporary societal representation of dementia, not least the voices of people living with dementia themselves and other forms of cultural text, including documentary and/or film and drama footage. All forms of knowledge are partial and constructed and situated, as is the argument that I am furthering here about an ascendant dementia prevention discourse and how that is, generally, noticeably absent from the talk of a small sample of those who are at the coal face of supporting a person who is already experiencing the (currently) inevitable demise of having a form of dementia.

2 All participant names are pseudonyms.

3 Current biomedical thinking is that the aetiology of vascular dementia is more closely aligned to other vascular conditions (for example, hypertension, cardiovascular disease, type 2 diabetes) and therefore diet and physical activity levels are more significantly linked to onset than in other types of dementia (Alzheimer's Society, 2013b; 2013c). While detailed discussion of the roots of the ascendancy of the individual responsibility discourse in dementia is beyond the scope of this article it is likely linked to this, alongside the ‘healthist’ emphasis in western cultures more generally.

## References

[b1] Adelman EE, Karlawish JHT (2003). Ignoring the controversies: newspaper reports on Alzheimer's disease treatments. Journal of the America Geriatrics Society.

[b2] Alleyne R (2011). A tipple ‘fights dementia’. Daily Telegraph.

[b3] Alzheimer's Society (2012). Dementia 2012: a National Challenge.

[b4] Alzheimer's Society (2013a). Dementia 2013: the Hidden Voice of Loneliness.

[b5] Alzheimer's Society (2013b). Am I at risk of developing dementia? Factsheet.

[b6] Alzheimer's Society (2013c). What is vascular dementia? Factsheet.

[b7] Armstrong J (2011). Tea cheers; green leaves fight dementia. The Mirror.

[b8] Barry N (2010). Dementia killed my husband… now I just want to help more Scots deal with that heartache. Sunday Express.

[b9] Bates C (2011). In a sequence of photographs some may find shocking, one man charts his mother's harrowing descent into Alzheimer's. Daily Mail.

[b10] Batsch NL, Mittelman MS (2012). World Alzheimer's Report 2012 Overcoming the Stigma of Dementia.

[b11] Beckford M (2011). Britain faces dementia catastrophe; Alzheimer's disease should be tackled as aggressively as Aids was in the eighties, warn charities. Daily Telegraph.

[b12] Bond J, Corner L, Graham R, Innes A, Archibald C, Murphy C (2004). Social science theory on dementia research: normal ageing, cultural representation and social exclusion. Dementia and Social Inclusion: Marginalised Groups and Marginalised Areas of Dementia Research, Care and Practice.

[b13] Borland S (2011). Can a low-fat diet stave off Alzheimer's?. Daily Mail.

[b14] Braun V, Clarke V (2006). Using thematic analysis in psychology. Qualitative Methods in Psychology.

[b15] Brooks L (2011). Comment: Alzheimer's is compelling in fiction because it's so cruel in life. The Guardian.

[b16] Bonnesen JL, Burgess EO (2004). Senior moments: the acceptability of an ageist phrase. Journal of Aging Studies.

[b17] Buckland D (2010). No one should be left to just fade away. Mirror.

[b18] Buckland D (2011). Fatties 80% more likely to develop Alzheimer's. Mirror.

[b19] Cannon E (2011). Keep fit to reduce the risk of dementia. Mail on Sunday.

[b20] Carlyle R (2010). Dementia Your questions answered; Brain power Who gets dementia, how can you avoid it and what are the treatments, asks Rachel Carlyle. The Times.

[b21] Carper J (2011a). What to eat to help prevent Alzheimer's; stock up on the food, vitamins and drinks that can reduce the risks of dementia. The Times.

[b22] Carper J (2011b). 100 Simple Things You Can Do To Prevent Alzheimer's and Age-Related Memory Loss.

[b23] Clarke JN (2006). The case of the missing person: Alzheimer's disease in mass print magazines 1991–2001. Health Communication.

[b24] Clarke JN, Everest MM (2006). Cancer in the mass print media: fear, uncertainty and the medical model. Social Science & Medicine.

[b25] Collin J, Hughes D (2011). The silent killer in media stories: representations of hypertension as health risk factor in French-language Canadian newspapers. Health, Risk and Society.

[b26] Conboy M (2002). The Press and Popular Culture.

[b27] Cook MS (2007). The Brain Wash: A Powerful, All-natural Program to Protect Your Brain Against Alzheimer's, Chronic Fatigue Syndrome, Depression, Parkinson's and Other Brain Diseases.

[b28] Corner L, Bond J (2004). Being at risk of dementia: fears and anxieties of older adults. Journal of Aging Studies.

[b29] Cutler SJ, Hodgson LG (1996). Anticipatory dementia: a link between memory appraisals and concerns about developing Alzheimer's disease. Gerontologist.

[b96] Daily Mail (2011). Rake some leaves to cut dementia risk. Daily Mail.

[b88] Daily Telegraph (2010). Don't overlook dementia. Daily Telegraph.

[b93] Daily Telegraph (2010). Stick to a high-fat diet to avoid Alzheimer's, say researchers. Daily Telegraph.

[b92] Daily Telegraph (2011). A pint of beer a day may keep dementia away. Daily Telegraph.

[b94] Daily Telegraph (2011). Weight gain link with dementia. Daily Telegraph.

[b95] Daily Telegraph (2011). Daily stroll helps ward off dementia, says study. Daily Telegraph.

[b30] Dementia Project http://www.dementiaproject.net.

[b31] Department of Health (2009). Living Well with Dementia: A National Dementia Strategy.

[b32] Dillner L (2010). Dr Luisa Dillner's guide to…: Signs of dementia. The Guardian.

[b89] Express (2010). Beetroot can fight dementia. Express.

[b86] Express (2011). Failing dementia patients. Express.

[b90] Express (2011). Vitamin C curbs dementia. Express.

[b33] Ferri CP, Prince M, Brayne C (2005). Global prevalence of dementia: a Delphi consensus study. Lancet.

[b83] Fletcher V (2010). Take a walk to keep dementia at bay. Express.

[b35] Gollust SE, Lantz PM (2009). Communicating population health: print news media coverage of type 2 diabetes. Social Science & Medicine.

[b36] Greening L, Greaves I, Greaves N, Jolley D (2009). Positive thinking on dementia in primary care: Gnosall memory clinic. Community Practitioner.

[b37] Hamilton S (2010). Brain disease on rise; Alzheimer's epidemic. Sunday Mirror.

[b38] Harding R, Peel E (2013). ‘He was like a zombie’: off-label prescription of antipsychotic drugs in dementia. Medical Law Review.

[b39] Holford P (2011). The Alzheimer's Prevention Plan: 10 Proven Ways to Stop Memory Decline and Reduce the Risk of Alzheimer's.

[b40] Hope J (2011a). Moderate drinking ‘can protect against dementia’. Daily Mail.

[b41] Hope J (2011b). Losing that spare tyre may help you avoid Alzheimer's. Daily Mail.

[b42] Jha A (2011). Obesity in middle age raises dementia risk by 300%. The Guardian.

[b43] Jolley DJ, Benbow SM (2000). Stigma and Alzheimer's disease: causes, consequences and a constructive approach. International Journal of Clinical Practice.

[b44] Jowett A, Peel E (2010). ‘Seismic cultural change?’ media representation of same-sex ‘marriage’. Women's Studies International Forum.

[b45] Kang S, Gearhart S, Bae H-S (2010). Coverage of Alzheimer's disease from 1984 to 2008 in television news and information talk shows in the United States: an analysis of news framing. American Journal of Alzheimer's Disease and Other Dementias.

[b46] Kastenbaum RJ, Gilmore A, Gilmore S (1988). ‘Safe death’ in the postmodern world. A Safer Death.

[b47] Kessler E-M, Schwender C (2012). Giving dementia a face? The portrayal of older people with dementia in German weekly news magazines between the years 2000 and 2009. Journals of Gerontology, Series B: Psychological Sciences and Social Sciences.

[b48] Kessler E-M, Bowen CE, Baer M, Froelich L (2012). Dementia worry: a psychological examination of an unexplored phenomenon. European Journal of Ageing.

[b49] Kirkman AM (2006). Dementia in the news: the media coverage of Alzheimer's disease. Australiasian Journal on Aging.

[b50] Kitzinger C, Peel E (2005). The de-gaying and re-gaying of AIDS: contested homophobias in lesbian and gay awareness training. Discourse & Society.

[b51] Knapp M, Prince M, Albanese E (2007). Dementia UK: a Report to the Alzheimer's Society on the Prevalence and Economic Cost of Dementia in the UK.

[b52] Laurance J (2011). Worried you'll get Alzheimer's? Then follow these seven steps. The Independent.

[b53] Lawton J, Ahmad N, Peel E, Hallowell N (2007). Contextualising accounts of illness: notions of responsibility and blame in white and South Asian respondents' accounts of diabetes causation. Sociology of Health & Illness.

[b54] Lawton J, Peel E, Douglas M, Parry O (2008). Shifting accountability: a longitudinal qualitative study of diabetes causation accounts. Social Science & Medicine.

[b55] Luengo-Fernandez R, Leal J, Gray A (2010). Dementia 2010: the Economic Burden of Dementia and Associated Research Funding.

[b56] Luft O (2011). ABC: i debuts with daily circulation of 133,472 in January. The Press Gazette.

[b57] Lupton D (1999). Editorial: health, illness and medicine in the media. Health.

[b58] Lyons AC (2000). Examining media representations: benefits for health psychology. Journal of Health Psychology.

[b59] McParland P (2012). An exploration into how the general public understand and respond to dementia. British Society of Gerontology 41st Annual Conference, Keele University.

[b60] MacRae F (2011). A vitamin B pill a day may ward off Alzheimer's. Daily Mail.

[b85] Mirror (2011). Dementia ‘A bomb ready to explode’; Warning. Mirror.

[b97] Mirror (2010). Stroll stops Alzheimer's. Mirror.

[b84] Morning Star (2011). Cancer and Alzheimer's most fear of diseases. Morning Star.

[b61] Murray M (2004). Critical Health Psychology.

[b62] Musso E, Wakefield S (2009). ‘Tales of mind over cancer’: cancer risk and prevention in the Canadian print media. Health, Risk and Society.

[b63] Naish J (2010). Do you think you're really losing your memory?. The Times.

[b64] Parry O, Peel E, Douglas M, Lawton J (2006). Issues of cause and control in patient accounts of type 2 diabetes. Health Education Research.

[b65] Peel E, Harding R (2013). ‘It's a huge maze, the system, it's a terrible maze’: dementia carers' constructions of navigating health and social care services. Dementia: The International Journal of Social Research and Practice.

[b66] Peel E, Parry O, Douglas M, Lawton J (2005). Taking the biscuit? A discursive approach to managing diet in type 2 diabetes. Journal of Health Psychology.

[b67] Potter J, Wetherell M (1987). Discourse and Social Psychology: Beyond Attitudes and Behaviour.

[b82] Revoir P (2011). The living death of Alzheimer's. Daily Mail.

[b68] Reynolds M (2011). Living a healthier life can halve risk from Alzheimer's. Express.

[b69] Rose N (2006). The Politics of Life Itself: Biomedicine, Power and Subjectivity in the Twenty-First Century.

[b70] Rich E (2011). ‘I see her being obesed!’: public pedagogy, reality media and the obesity crisis. Health.

[b71] Riggs DW, Castañeda L, Campbell S (2005). ‘Proving the case’: psychology, subjectivity and representations of lesbian and gay parents in the media. News and Sexuality: Media Portraits of Diversity.

[b72] Seale C (2002). Media and Health.

[b73] Sontag S (1988). AIDS and its Metaphors.

[b74] Smith R (2010). Chemical cosh will be cut for dementia sufferers. Daily Telegraph.

[b87] Sunday Mirror (2011). Reagan had Alzheimer's ‘in office’. Sunday Mirror.

[b75] Sweeting H, Gilhooly M (1997). Dementia and the phenomena of social death. Sociology of Health and Illness.

[b91] The Guardian (2011). Drinking green tea may help ward off Alzheimer's. The Guardian.

[b76] Taylor JA (2012). http://www.amazon.co.uk/review/R2JOAEA57US87Q/ref=cm_cr_pr_viewpnt#R2JOAEA57US87Q.

[b77] Victoroff J (2004). Saving Your Brain: The Revolutionary Plan to Boost Brain Power, Improve Memory and Protect Yourself against Aging and Alzheimer's.

[b78] Wainwright M (2010). Why walking nine miles a week could save you from dementia. The Guardian.

[b79] Willey J (2011a). How staying slim slashes risk of dementia. Express.

[b80] Willey J (2011b). How a little exercise in middle age could help beat dementia. Express.

[b81] Williamson JML, Skinner CI, Hocken DB (2011). Death and illness as depicted in the media. International Journal of Clinical Practice.

